# Predictors of psychological distress among Afghan and Syrian refugee women in Houston, Texas

**DOI:** 10.3389/fgwh.2026.1830947

**Published:** 2026-07-09

**Authors:** Jenna Zamil, Isaiah Olumeko, Samuel Ofili, Sai Cheruvu, Paroma Arefin, Fatin A. Atrooz, Farzana Ahmad, Samina Salim

**Affiliations:** 1 School of Medicine, Baylor College of Medicine, Houston, TX, United States; 2 Department of Pharmaceutical Health Outcomes and Policy, College of Pharmacy, University of Houston, Houston, TX, United States; 3 Department of Pharmacological and Pharmaceutical Sciences, College of Pharmacy, University of Houston, Houston, TX, United States

**Keywords:** Afghan, mental health, refugee women, stress, Syrian, trauma

## Abstract

Refugee mental health is shaped by a complex interplay of pre-migration trauma, migration-related hardship, and post-migration stressors. Afghan and Syrian refugees constitute two of the largest displaced populations resettled in the United States, yet their mental health needs remain understudied particularly among women, who may face layered vulnerabilities linked to their gender-based roles within the family. Houston, Texas, hosts sizeable communities of both groups, creating a unique context for examining culturally specific expressions of distress and the predictors of post-migration stress. This study compares mental health profile of Afghan and Syrian refugee women living in the Houston metropolitan area and identifies demographic and sociocultural factors associated with psychological distress. Mental health in both groups was assessed using two psychometrically validated measures: the Afghan Symptoms Checklist (ASC) and the Refugee Post-Migration Stress (RPMS) scale. Sociodemographic data was collected and included in a multiple linear regression model to identify predictors of psychological symptoms. Differences in psychological distress patterns were observed between Afghan and Syrian refugee women; however, these differences were not statistically significant in adjusted models. Perceived discrimination and social strain emerged as significant predictors of psychological distress across multiple models. These findings highlight the need for culturally tailored community-engaged mental health interventions that address both the universal challenges of resettlement and the unique cultural frameworks shaping psychological state among refugee women.

## 1 Introduction

The mental health of refugees is multifaceted, shaped by pre-migration trauma, the challenges of forced migration, as well as the pressures of resettlement (1). Prior to displacement, many refugees experience several traumas related to war, political instability, persecution, loss of loved ones, and exposure to direct or indirect violence (2). These early adversities are only the first layer of risk. Upon resettlement, refugees frequently encounter stressors such as social isolation, discrimination, unemployment or underemployment, financial strain, language barriers, and difficulties navigating unfamiliar systems of healthcare, education, and social support (3, 4). These overlapping stressors collectively influence mental health outcomes across refugee populations (5). Among these groups, Afghan and Syrian refugees represent two of the largest and most vulnerable displaced communities globally (6). Each group carries unique cultural, historical, and social experiences that shape their mental health. For Syrian refugees, prolonged civil war, and conflict resulting in protracted displacement have resulted in high rates of psychological trauma (7). Studies indicate that post-migration stress, particularly financial insecurity and social isolation, is strongly associated with a lower quality of life. For example, a Swedish study showed that limited social networks, perceived loneliness, and economic hardship significantly contributed to poorer mental well-being among Syrian refugees (8). With limited resources to address the mental health needs of the Syrian population due to factors like lack of mental healthcare access and loss of psychiatric hospital infrastructure in Syria, addressing the mental health of Syrian refugees in the United States is critical (7).

Afghan refugees, by contrast, represent one of the longest-displaced populations in the world, having endured decades of conflict across generations. Research among Hazara Afghan refugees in Australia documented clinically significant depressive symptoms in more than 21% of participants, with women experiencing nearly three times the prevalence in men, underscoring gender-specific vulnerability (9). A systematic review also found that Afghan women are particularly vulnerable when it comes to mental health risks (10). Cultural expectations around emotional endurance and collective suffering may further complicate Afghan women's willingness or ability to disclose distress.

Despite both groups experiencing significant adversity, Afghan and Syrian refugees differ markedly in cultural frameworks that shape the perception and expression of psychological distress. A systematic review comparing the two groups found that Syrian refugees were less likely to interpret psychological distress as pathological, instead viewing emotional suffering as an expected part of life (11). Afghan refugees, on the other hand, exhibited strong stigma surrounding formal mental health care, including therapy and psychiatric support services (11). These cultural beliefs can mask symptoms, delay help-seeking, and create challenges for healthcare providers attempting to identify mental health needs. Given these complexities, direct engagement with refugee communities is essential for understanding their psychological distress especially among women who may face compounding stressors such as caregiving responsibilities, limited mobility, social isolation, and gender-specific trauma (12–14). One study among Afghan and Syrian refugees in Turkey found that female gender was a predictor of increased psychological distress (15). Houston is a particularly important setting for this work. With an estimated 5,000–15,000 Afghan refugees resettled as of 2025 and approximately 20,000 Syrian refugees as of 2021, the city is one of the most demographically significant refugee hubs in the United States (3, 16). This growing population presents a critical opportunity to strengthen culturally informed mental health initiatives.

This study is conceptually grounded in the social determinants of health framework and post-migration stress models, which posit that structural and environmental stressors such as discrimination, economic hardship, and social isolation interact with individual-level factors to influence mental health outcomes among refugee populations. The primary aim of this study was to identify demographic and post-migration stress-related predictors of psychological distress among Afghan and Syrian refugee women. In the present study, psychological distress is used as the primary construct to describe emotional, cognitive, behavioral, and somatic manifestations of mental health difficulties measured by the Afghan Symptom Checklist. We hypothesized that higher levels of post-migration stressors, particularly perceived discrimination and social strain, would be associated with increased psychological distress.

## 2 Methods

### 2.1 Study design and setting

This cross-sectional study was conducted in the Greater Houston area (Harris County) between 2022 and 2023. The study was approved by the Institutional Review Board (IRB) Committee for the Protection of Human Subjects at the University of Houston (STUDY00002929). The current study builds on our previous work examining gender differences (3). A subset of the Syrian refugee sample was included in this study by focusing exclusively on women and comparing Houston-based Afghan and Syrian refugee groups, addressing a distinct new research question.

### 2.2 Participants

Syrian and Afghan refugee women resettled in Harris County were recruited using a snowball sampling method. Eligibility criteria included being a Syrian or Afghan refugee woman aged 18 years or older. Prior to participation, the research team explained the study objectives and obtained informed consent from all participants.

### 2.3 Data collection and procedures

Survey instruments were translated into Arabic and Dari and administered via the secure UH-REDCap platform. Participants accessed the survey through a REDCap link and provided electronic consent. Surveys could be completed independently or with assistance from a trained Arabic/Dari-speaking research team members. Participants received a $20 gift card upon completion.

### 2.4 Variables and measures

Data were collected on age, education, relationship status, household size, health insurance, employment status, and monthly income. Health-related measures included body mass index (BMI) and chronic disease prevalence (diabetes mellitus, hypothyroidism, asthma, and irritable bowel syndrome), as these measures are reported to influence mental health outcomes (17–19).

### 2.5 Psychological and post-migration measures

Psychological distress was assessed using the Afghan Symptom Checklist (ASC), a 22-item instrument measuring distress across three domains: sadness with social withdrawal (Jigar Khun), ruminative sadness (Fishar), and aggression/reactivity. Post-migration stressors were assessed using the Refugee Post-Migration Stress Scale (RPMS), a 21-item instrument measuring perceived discrimination, economic strain, social strain, and host-country adaptation challenges.

The Afghan Symptom Checklist (ASC) was selected for its ability to capture culturally grounded and somatic expressions of distress, while the Refugee Post-Migration Stress Scale (RPMS) was used to assess structural and environmental stressors associated with resettlement. Together, these instruments allow for examination of both internal symptom expression and external stress exposures. Although the ASC was originally developed for Afghan populations and the RPMS validated among Syrian refugees, both instruments were applied across groups to enable comparative analysis. Furthermore, ASC was previously used and exhibited high reliability in our previous studies (3, 20) conducted in the Arab population including Syrian group. In our study, the ASC was used to capture broader dimensions of psychological distress, including somatic and affective symptoms that are not exclusively culture-bound. Furthermore, as detailed in our previous publication (3) some terms in ASC were replaced with culturally appropriate phrases during Arabic translation. Notably, the ASC was originally developed for Afghan populations, it was applied across both Afghan and Syrian refugee groups to facilitate comparative analyses. However, culturally specific constructs such as *Jigar Khun* and *Fishar* may not be interpreted identically across populations. Therefore, intergroup comparisons involving ASC-derived symptom domains were interpreted cautiously and the same is recommended.

The RPMS is specifically designed to assess stressors encountered in the host country, including perceived discrimination, social isolation, and loss related to the home country (21). Importantly, the RPMS is grounded in socio-economic and political stressors associated with the resettlement context rather than culturally specific idioms of distress. As such, its constructs are intended to be broadly applicable across refugee populations experiencing post-migration challenges in different host settings. Since contextual adaptation is essential when applying such instruments across settings, therefore, RPMS was reviewed and tailored to reflect U.S. specific conditions, including resettlement experiences. Given that the instrument measures structural and environmental stressors rather than culturally bound psychological constructs, its use in a U.S.-based refugee population is appropriate and consistent with its intended purpose.

### 2.6 Translation and cultural adaptation

Survey instruments were translated into Arabic and Dari using a standardized multi-step process including forward translation, backward translation, reconciliation, and pilot testing to ensure clarity and cultural appropriateness. Linguistic translation was conducted in accordance with established validation guidelines (22, 23). The process consisted of four steps. *First*, forward translations (English to Arabic and English to Dari) were completed by bilingual members of the research team. Two team members are native Arabic speakers and two are native Dari/Pashto speakers. *Second*, backward translations (Arabic to English and Dari to English) were performed by two bilingual faculty members at the University of Houston, Houston, TX, and the Western University College of Pharmacy, Pomona, CA. *Third*, the back-translated versions were compared with the original questionnaires by the primary investigator, and any discrepancies were resolved through iterative refinement of the Arabic and Dari expressions until no further discrepancies remained (22). *Fourth*, the translated versions were pilot-tested with five Arabic- and Dari-speaking respondents to assess clarity, comprehension, and acceptability (23).

*Afghan Symptoms Checklist (ASC)*- The ASC instrument was originally developed and validated in Kabul, Afghanistan, the ASC identifies culturally relevant indicators of psychological distress in conflict and post-conflict contexts (24). Notably, some ASC subscales (e.g., *jigar khun*, *fishar*) are culturally rooted constructs, others capture more universal expressions of distress. The Arabic version of the ASC has previously demonstrated excellent reliability in our study of Arab American refugees and immigrants resettled in Houston, Texas (3). The ASC is a 22-item instrument that assesses feelings and experiences over the past two weeks. Items are rated on a 5-point scale from 1 (“never”) to 5 (“everyday”), yielding total scores ranging from 22 to 110, with higher scores reflecting greater distress. The scale comprises three subscales that show high reliability in our previously reported publication (3):

*1. Sadness with social withdrawal and somatic distress (Jigar Khun)* – characterized by social isolation, frequent crying, loss of appetite, experiencing nightmares, headaches, and profound hopelessness. The fourteen-item *Jigar Khun* subscale demonstrated excellent internal consistency (Cronbach's *α* = 0.905) (Supplementary Table S5).
*2. Ruminative sadness without social isolation or somatic distress (Fishar)* – marked by extreme perceived stress and difficulties with concentration. The two-item Fishar subscale demonstrated modest internal consistency (Cronbach's *α* = 0.60). The inter-item correlation was moderate and statistically significant (*r* = 0.42, *p* < 0.001). Given that Cronbach's alpha may underestimate reliability for two-item scales (25), the Spearman-Brown coefficient was also calculated (*ρ* = 0.59), indicating moderate internal consistency (Supplementary Table S5). Given that the scale consists of only two items, reliability estimates are inherently constrained; therefore, this coefficient is considered acceptable for a brief subscale (25, 26). Consequently, Fisher subscale data requires cautious interpretation.
*3. Aggression or stress-induced reactivity* – reflected in quarrels, irritability, and, in some cases, physical aggression such as striking children. The four-item aggression subscale demonstrated low internal consistency (Cronbach's *α* = 0.409) (Supplementary Table S6), consequently, the subscale data requires cautious interpretation.

The modest reliability of the Fishar subscale and the low reliability of the Aggression subscale may increase measurement error and attenuate observed associations, potentially resulting in conservative estimates of effect sizes. Therefore, findings involving these domains should be interpreted cautiously.

*The Refugee Post-Migration Stress (RPMS) scale-* RPMS was used to assess stressors experienced by refugee women following resettlement in Houston (27). The instrument consists of 21 items grouped into seven domains of post-migration stress: [1] perceived discrimination, [2] lack of host country–specific competencies, [3] material and economic strain, [4] loss of home country, [5] family and home country concerns, [6] social strain, and [7] family conflicts. Responses are rated on a 5-point Likert scale ranging from 1 (never) to 5 (very often). In the present analysis, we focused on four domains: perceived discrimination, lack of host country specific competencies, material and economic strain, and social strain, while excluding family conflict, family/home country concerns, and loss of home country. This decision was guided by both conceptual and methodological considerations. *First*, our sample includes Afghan and Syrian refugees, whose migration trajectories and conditions in their countries of origin are substantially different. Domains such as loss of home country and family/home country concerns are highly context-dependent and may not be directly comparable across these different populations that could bias estimates and limit interpretability. *Second*, the family conflict domain measures intra-household dynamics that are influenced by culturally specific norms and reporting patterns, which may vary considerably across populations. This raises concerns about the validity of cross-group comparisons. In contrast, the selected domains reflect more consistent stressors related to the host country environment, which are more comparable across refugee groups and more directly aligned with the study objectives. The perceived discrimination domain consists of four items assessing experiences such as discrimination by American authorities, discrimination in school or at work, feeling disrespected due to one's national background, and being the target of racist remarks. The lack of host country-specific competences domain includes three items assessing difficulties communicating in English, understanding how everyday activities in the United States work (e.g., shopping, purchasing tickets, traveling), and understanding documents or forms from U.S. authorities. The material and economic strain domain comprises three items assessing worries about an unstable financial situation, frustration from being unable to support oneself financially, and worries about debts. The social strain domain consists of three items assessing feelings of exclusion or isolation in American society, frustration due to loss of status in American society, and frustration from being unable to utilize one's skills and competencies in the United States. For each domain, item scores were summed to create a composite variable, with higher scores indicating greater levels of the construct. The possible score ranges were; perceived discrimination (4–20 points), lack of host country-specific competences (3–15 points), material and economic strain (3–15 points), and social strain (3–15 points). The four domains included in the analysis showed a moderate to high reliability (Cronbach's *α* = 0.67–0.87) (Supplementary Table S6).

### 2.7 Statistical analysis

Descriptive statistics were used to summarize the demographic and general health characteristics of the participants. Comparisons between sociodemographic characteristics of Syrian and Afghan women were conducted using independent t-tests for continuous variables (presented as means and standard deviations) and Chi-square tests for categorical variables (presented as frequencies and percentages). A *p*-value of < 0.05 was considered statistically significant. All analyses were performed using SAS version 9.4.

#### 2.7.1 Statistical modeling

Aggression, Jigar Khun, and Fishar scores were modeled using linear regression. Independent variables were selected using a combination of statistical and theory-driven approaches. Univariate analyses (*p* ≤ 0.20) were used as an initial screening step; however, final model variables were selected based on prior literature and their clinical and theoretical relevance to the outcomes of interest. Participants with missing values on variables included in a given analysis were excluded from the analysis. Missing data was primarily driven by incomplete responses to ASC subscale items, RPMS domain items and selected demographic variables (e.g., income). Importantly, missing data patterns did not differ substantially between Afghan and Syrian participants, suggesting that differential missingness by refugee group is unlikely to have introduced systematic bias. We observed no statistically significant differences between the included and excluded groups regarding age (*p* = 0.556–0.853), education (*p* = 0.402–0.818), or family income (*p* = 0.134–0.693). A significant difference was noted in the distribution of refugee group (*p* = 0.002 for aggression), indicating that nationality was associated with the likelihood of having complete data. To account for this, nationality was strictly controlled for as a covariate in all final models. These findings confirm that, for the variables requested, the analyzed cohorts remain representative of the broader study population (Supplementary Table S7).

*Predictors of Aggression symptoms-* The primary outcome, the Aggression score, was derived from four items of the Afghan Symptoms Checklist (ASC), a 22-item, 5-point Likert scale instrument. The Aggression score is a continuous variable ranging from 4 to 20, with higher scores indicating greater aggression. Considering prior literature linking physical health and mental health outcomes (17, 18, 28), the following independent health-related variables: age, education, employment, marital status, family income, insurance, living with partner, partner's education, cigarette smoking, hookah smoking, household number, body mass index, diabetes mellitus, hypertension, hypothyroidism, asthma, and irritable bowel syndrome, were added into the multivariable model. Annual income was grouped as < $10,000 and ≥ $10,000 because $10,000 represents a policy-relevant financial threshold used in refugee asset-building programs, such as the Individual Development Accounts program, to identify individuals with limited economic resources. The final multivariable linear regression model (Table 2) assessed the independent associations of aggression with refugee origin (Afghan vs. Syrian), selected post-migration stress domains (Perceived Discrimination, Material and Economic Strain, Lack of Host Country Competence, and Social Strain from the Refugee Post-Migration Stress scale), and key demographic and health-related covariates. Model assumptions were checked through residual analysis.

*Predictors of Jigar Khun symptoms*- The Jigar Khun domain, one of the three symptom clusters in the Afghan Symptom Checklist (ASC), was used to assess sadness with social withdrawal and somatic distress over the preceding two weeks. This domain includes 14 items that ask respondents how often they experienced specific symptoms during that period, such as crying, loss of appetite, difficulty falling asleep, feelings of hopelessness or extreme hopelessness, social withdrawal, sadness, irritability, being easily startled (e.g., by sudden noises), intrusive or distressing memories, painful or heavy sensations in the heart, nightmares, and difficulties with memory, among others. Each item is rated on a five-point Likert scale (1 = never, 2 = 1 day/week, 3 = 2–3 days/week, 4 = 4–5 days/week, 5 = every day). Item scores are summed to create a composite Jigar Khun score ranging from 14 to 70, with higher scores indicating greater severity of sadness, social withdrawal, and somatic distress. A multivariable linear regression analysis (Table 3) was performed with the Jigar Khun score as the dependent variable to assess its associations with perceived discrimination, lack of host country-specific competences, material and economic strain, social strain, refugee status, education, family income, living with a partner, insurance status, age, BMI, and household size.

*Predictors of Fishar (Ruminative Sadness) symptoms-* Fishar Score, representing ruminative sadness, was measured using two items from the Afghan Symptom Checklist (ASC): “During the last 2 weeks, how many times have you had trouble concentrating?” and “During the last 2 weeks, how many times have you had difficulty meeting your responsibilities at home or at work because you had a sense of extreme hopelessness?”. Each item was rated on a 5-point Likert scale (1 = Never to 5 = Very Often). Scores from these two items were added up to create a composite Fishar score ranging from 2 to 10, with higher scores indicating greater levels of ruminative sadness. The Fishar score was modeled as the dependent variable in a two-step approach. First, we conducted univariate linear regression analyses to screen for potential predictors of the Fishar score. The final multiple linear regression model assessed the independent associations of aggression with refugee origin (Afghan vs. Syrian), selected post-migration stress domains (Perceived Discrimination, Material, and Economic Strain, Lack of Host Country Competence, and Social Strain from the Refugee Post-Migration Stress scale), and key demographic covariates including age, BMI, insurance, education, and family income.

## 3 Results

### 3.1 Sociodemographic characteristics

Table 1 presents key differences between Syrian and Afghan participants. Syrian participants were older than Afghan participants (40.4 vs. 32.9 years, *p* < 0.0001). They were more likely to live with a partner (83.9% vs. 61.2%, *p* = 0.0058) and had higher rates of insurance coverage (82.3% vs. 56.9%, *p* = 0.002). Afghan participants more frequently reported annual family income below $10,000 (76.2% vs. 55.9%, *p* = 0.0179). Household size was slightly larger among Syrians (4.0 vs. 3.1, *p* = 0.033). Differences were also observed in clinical measures. Syrians had higher mean BMI (29.4 vs. 24.8, *p* < 0.0001) and a greater prevalence of hypertension (14.1% vs. 3.0%, *p* = 0.0223). Psychosocial measures showed notable variation between groups. Afghan participants had higher ASC scores (39.55 vs. 33.63, *p* = 0.0145) and higher levels of Jigar Khun (27.65 vs. 21.89, *p* = 0.0016), while Syrian participants reported higher perceived discrimination (6.48 vs. 4.88, *p* = 0.0059). Other characteristics are presented in Supplementary Table S1.

**Table 1:** Key characteristics and psychosocial measures by refugee group.

Characteristic	Total (*N* = 131)	Syrian (*n* = 64)	Afghan (*n* = 67)	*p*-value
Socio-demographics				
Age, years: mean (SD)	36.6 (9.5)	40.4 (10.1)	32.9 (8.7)	<0.0001
Marital Status, *n* (%)				0.021
Married	101 (78.9%)	49 (80.3%)	52 (77.6%)	
Separated/Divorced/Widowed	5 (3.9%)	5 (8.2%)	0 (0.0%)	
Single (Never Married)	22 (17.2%)	7 (11.5%)	15 (22.4%)	
Living with Partner (Yes), *n* (%)	93 (72.7%)	52 (83.9%)	41 (61.1%)	0.006
Family Income < $10,000, *n* (%)	81 (66.4%)	33 (55.9%)	48 (76.2%)	0.018
Insurance (Yes), *n* (%)	88 (69.3%)	51 (82.3%)	37 (56.9%)	0.002
Household Number, mean (SD)	3.6 (2.3)	4.0 (2.2)	3.1 (2.3)	0.033
Clinical Factors				
BMI (kg/m2): mean (SD)	27.1 (5.9)	29.4 (6.5)	24.8 (5.2)	<0.0001
Hypertension (HTN), *n* (%)	11 (8.4%)	9 (14.1%)	2 (3.0%)	0.022
Psychosocial Measures				
ASC Total Score: mean (SD)	36.63 (13.93)	33.63 (12.04)	39.55 (15.07)	0.015
Perceived Discrimination: mean (SD)	5.51 (2.48)	6.48 (2.74)	4.88 (2.10)	0.006
Jigar Khun: mean (SD)	24.82 (10.60)	21.89 (8.20)	27.65 (11.87)	0.002

### 3.2 Multivariable linear regression model for aggression

Mean (SD) scores for ASC subscales and RPMS domains were calculated and compared between groups. Afghan participants demonstrated higher mean ASC scores, while Syrian participants reported higher RPMS perceived discrimination scores; however, these differences were not statistically significant. Table 2 presents the results of the overall multivariable linear regression model.

**Table 2:** Adjusted multivariable linear regression analysis of predictors associated with aggression.

Predictor	Estimate	*p*-value	95% CI
Refugee Group (Ref: Afghan)			
Syrian	0.8068	0.1003	[−0.160, 1.774]
Perceived discrimination	0.1953	0.0146***	[0.040, 0.351]
Lack of host country-specific competencies	0.014	0.7874	[−0.090, 0.118]
Economic strain	0.0007	0.9904	[−0.116, 0.117]
Social strain	0.0884	0.1716	[−0.039, 0.216]
Age	0.0309	0.2227	[−0.019, 0.081]
BMI	−0.0371	0.2928	[−0.107, 0.033]
Insurance (Ref: YES)			
NO	0.2566	0.6093	[−0.743, 1.256]
HTN (Ref: YES)			
NO	2.0377	0.0382***	[0.114, 3.961]
Education (Ref: < High School)			
High school	−0.2399	0.6313	[−1.236, 0.756]
> High school	0.5237	0.2777	[−0.433, 1.481]
Family Income (Ref: < $10,000)			
>$10,000	−0.0318	0.9433	[−0.922, 0.858]

Statistical Significance ***.

Perceived discrimination emerged as a statistically significant positive predictor of aggression. For every one-unit increase in perceived discrimination, aggression score was estimated to increase by 0.1953 units (*p* = 0.0146, 95% CI [0.040, 0.351]). This suggests that higher levels of perceived discrimination are associated with higher aggression scores.

Additionally, a significant association was found for hypertension. Participants who did not have hypertension were estimated to have 2.0377 units higher aggression scores than those who had hypertension (*p* = 0.0382, 95% CI [0.114, 3.961]). The association between hypertension and aggression should be interpreted cautiously due to the small number of participants reporting hypertension.

Other factors, including refugee group status (Syrian vs. Afghan), lack of host country competence, economic strain, social strain, age, BMI, insurance status, education levels, and family income, were not found to be statistically significant predictors of aggression in this model.

### 3.3 Multivariable linear regression model for Jigar Khun

In the adjusted Multivariable Linear Regression model of Jigar Khun (Table 3), not living with a partner (*β* = 7.06; 95% CI: 0.68–13.44; *p* = 0.031) was significantly associated with a 7.06-point higher sadness with social withdrawal and somatic distress score (Jigar Khun score), when other variables are kept constant. Additionally, one unit increase in perceived discrimination (*β* = 1.06; 95% CI: 0.22–2.10; *p* = 0.046) was associated with a 1.06-point increase in the Jigar Khun score, and one unit increase in social strain (*β* = 1.02; 95% CI: 0.16–1.87; *p* = 0.021) was associated with a 1.02-point increase in the Jigar Khun score. All other variables were not statistically significant.

**Table 3:** Adjusted multivariable linear regression analysis of predictors associated with sadness with social withdrawal and somatic distress.

Variable	Estimate	CI	*P*-value
Refugee			
Syrian	Reference		
Afghanistan	5.937	−0.680–12.554	0.078
Education			
<High School	Reference		
High School	4.019	−2.430–10.470	0.217
>High School	4.622	−1.898–11.142	0.161
Family Income			
< $10,000	Reference		
>$10,000	−2.279	−8.303–3.745	0.452
Living with Partner			
Yes	Reference		
No	7.059	0.680–13.438	0.031***
Insurance			
Yes	Reference		
No	−1.123	−7.774–5.528	0.736
Age	0.047	−0.226–0.320	0.730
BMI	−0.265	−0.712–0.181	0.239
Household Number	0.022	−1.111–1.154	0.970
Perceived Discrimination	1.059	0.220–2.098	0.046***
Lack of Host Country-Specific Competences	0.598	−0.097–1.293	0.090
Material and Economic Strain	−0.179	−0.941–0.583	0.641
Social Strain	1.017	0.161–1.873	0.021***

Statistical significance ***.

### 3.4 Multivariable linear regression model for Fishar

In the adjusted Multivariable Linear Regression model for the Fishar score (Table 4), social strain was significantly associated with higher Fishar scores, indicating greater ruminative sadness (B = 0.137, *p* = .037, 95% CI [0.009, 0.266]). Participants with higher than a high school education reported higher Fishar scores compared to those with less than a high school education (B = 1.026, *p* = .008, 95% CI [0.268, 1.785]). No other demographic, socioeconomic, or psychosocial covariates, including refugee group, perceived discrimination, lack of host country–specific competencies, economic strain, age, BMI, insurance status, or family income,showed statistically significant associations with Fishar score. Model fit statistics for all regression models (including R^2^, F-statistics, and Root MSE) are provided in Supplementary Table S2, and multicollinearity diagnostics indicated no evidence of problematic collinearity among predictors (all VIFs < 5; see Supplementary Tables S3–S5).

**Table 4:** Adjusted multivariable linear regression analysis of predictors associated with fishar score.

Predictor	Estimate	*p*-value	95% CI
Refugee Group (Ref: Afghan)			
Syrian	−0.039	0.914	[−0.762, 0.683]
Perceived discrimination	0.067	0.412	[−0.095, 0.230]
Lack of host country-specific competencies	0.061	0.247	[−0.043, 0.165]
Economic strain	0.034	0.556	[−0.080, 0.147]
Social strain	0.137	0.037***	[0.009, 0.266]
Age	0.009	0.607	[−0.027, 0.046]
BMI	−0.028	0.337	[−0.087, 0.030]
Insurance (Ref: YES)			
NO	−0.415	0.289	[−1.187, 0.357]
Education (Ref: < High School)			
High school	0.518	0.205	[−0.288, 1.325]
> High school	1.026	0.008***	[0.268, 1.785]
Family Income (Ref: < $10,000)			
>$10,000	−0.032	0.930	[−0.738, 0.675]

Statistical Significance ***.

## 4 Discussion

Overall, our results show several significant predictors of psychological distress among Afghan and Syrian refugee women that can be used to guide the development of culturally adapted intervention for these groups. *First*, our study found that not living with a partner predicted greeted sadness with social withdrawal and somatic distress. A previous study among Syrian refugee women in Jordan who were heads of their households found that over 90% had experienced death threats (29). In this cohort of which 50.5% were widowed, over 90% reported symptoms of emotional distress (29). Another survey among Syrian refugees in Sweden found that both women and refugees who were widowed or divorced reported higher rates of mental disorders (anxiety, depression, low subjective well-being and/or PTSD) (21). Widowed or divorced women, thus, may constitute a subgroup of Afghan and Syrian refugees in need of more targeted interventions for mental health.

Fishar subscale results should be considered as exploratory rather than confirmatory due to the low Croncbach's alpha value. Having acknowledged this, it is important to mention that participants with higher than a high school education level reported greater Fishar scores. One possible explanation is underemployment; however, this was not directly measured and should be explored in future studies. One study conducted among refugees in Alberta, Canada found that refugees were underemployed compared to their education level; furthermore, they experienced greater unemployment than people born in Canada regardless of education level (30). 75% of refugees surveyed could not return to a “managerial/professional” profession after moving to Canada, and women were less likely than men to work in a similar area to their pre-migration job (30). Another study among Afghan refugees in Germany found that several factors contribute to underemployment of Afghan and Syrian refugees; these include language barriers, lost certification or education documentation, education credentials that do not transfer to the new country, and lack of jobs in their exact field (4). A different study among Afghan, Syrian, Iraqi, and Iranian refugees in Austria found less overqualification among women, but lower pay (31). With fluctuation in the American economy following the COVID-19 pandemic at the time of our survey and onward into the present, further research should evaluate whether Syrian refugee women are underemployed and/or undercompensated for their education level. Furthermore, whether underemployment is a predictor of psychological distress should also be evaluated.

Higher Fishar scores were also associated with greater social strain. This result is consistent with previous research, and factors contributing to social strain are also outlined in previous research (8). One systematic review emphasizes the potential for isolation when Afghan refugees resettle in Western countries, where they are less likely to encounter Afghan communities than in countries that are geographically closer (32). This review evaluated 7 studies in Europe on evidence-based interventions into Afghan refugee mental health, with the ultimate end goal of evidence-based interventions (EBI) into mental health (32). Our results can be used to tailor EBI in the US to emphasize factors of social strain, like isolation. A previous study in Norway suggested that among Afghan refugees, a feeling of connectedness to Norwegian society is associated with better mental health (33). With our results guiding interventions to address isolation, better outcomes can be achieved in the US.

Furthermore, we found a nearly linear positive correlation between perceived discrimination and Jigar Khun score. A previous study among Afghan refugees in Northern California found that perceived discrimination was associated with psychological distress (34). Similar results are observed among Syrian refugees in Norway (35). With our results emphasizing this trends among Afghan and Syrian refugee women in the same city, a more precise understanding of how perceived discrimination affects mental health among women in these populations can guide interventions.

Our study found that both BMI and hypertension prevalence were higher among Syrian refugees than Afghan Refugees. A study among Syrian refugees in Northen Jordan found that nearly 40% of participants had hypertension upon biological testing, but only 31.3% of such participants self-reported hypertension (36). Over 80% of participants were overweight or obese (36). While our study found no significant difference between Afghan and Syrian refugee women reporting diabetes, this previous study in Jordan found that Syrian refugee women had a higher prevalence of diabetes and being overweight or obese than Syrian refugee men (36). As our study surveys women exclusively, further inquiry should evaluate whether the same sex discrepancy is observed among Afghan refugee populations. Additionally, future research should investigate why Syrian refugees display higher rates of hypertension, and access to care for hypertension should be emphasized alongside interventions for mental health. The observed association between the absence of hypertension and higher aggression scores should be interpreted with caution. The number of participants with hypertension was small, particularly among Afghan participants, which may result in unstable estimates and sensitivity to outliers. This finding may therefore reflect sample limitations rather than a true underlying relationship. Further discrepancies we observed between Afghan and Syrian refugee women were related to socioeconomic status. Our study found that more Syrian refugee women reported higher household income and health insurance coverage; future research should work to outline barriers to healthcare access in these populations and especially among Afghan refugees.

### 4.1 Limitations and future perspectives

This study has several limitations. [1] The sample size was modest, limiting statistical power. [2] Snowball sampling may introduce selection bias and limit generalizability. Specifically it may introduce network homogeneity bias, whereby participants are more likely to recruit individuals with similar backgrounds, social networks, and lived experiences. This may reduce sample diversity and limit representativeness, particularly in heterogeneous refugee populations. As a result, findings may reflect characteristics of specific social networks rather than the broader population (37, 38). [3] The cross-sectional design precludes causal inference. [4] Afghan and Syrian refugees in the U.S. may have differing legal pathways (e.g., refugee status, asylum, humanitarian parole), which can influence access to healthcare, insurance coverage, and social services. [5] Key variables such as trauma severity, acculturation, and English proficiency were not measured. [6] We did not apply formal corrections for multiple testing across the three regression models. Although each model examined a distinct, predefined domain of psychological distress, the possibility of inflated Type I error cannot be excluded, particularly for findings with borderline statistical significance. [7] Approximately 46% of the sample was excluded from two of the three regression models due to missing data, resulting in reduced analytic sample sizes and lower observations-per-predictor ratios. This may increase the risk of overfitting and limit the stability of model estimates. Another limitation relates to the use of the ASC among Syrian refugee women. Although the Arabic version has demonstrated good reliability in prior studies, formal measurement invariance between Afghan and Syrian populations was not established. Consequently, observed differences in ASC-derived domains, including *Jigar Khun*, *Fishar*, and *Aggression*, may reflect both true differences in psychological distress and cultural differences in symptom interpretation or expression. Future studies should consider the use of multiple imputation methods to address missing data more robustly. [8] Given the relatively small sample size in relation to the number of predictors, there is a potential risk of overfitting, which may limit the generalizability of the model. [9] While Arabic-translated ASC has been demonstrated, this does not establish construct validity or measurement equivalence across cultural groups, thus, formal measurement invariance testing was not conducted, cross-cultural construct equivalence between Afghan and Syrian participants cannot be assumed and group-level comparisons involving ASC scores should be interpreted with caution. [10] An important limitation is the modest internal consistency of the Fishar subscale (*α*=0.60) and the low internal consistency of the Aggression subscale (*α*=0.409). Reduced reliability may introduce measurement error, decrease statistical power, and attenuate true associations between post-migration stressors and psychological distress outcomes. Consequently, significant findings may underestimate the magnitude of underlying relationships, whereas non-significant findings should be interpreted with caution. Future studies should further evaluate the psychometric properties of these subscales, explore culturally adapted item expansion, and assess measurement invariance across refugee populations. Self-reported data may be subject to recall and social desirability bias. Nonetheless, we propose several statistically significant predictors of psychological distress among Afghan and Syrian refugee women with the potential to impact mental health interventions in these populations. Our findings suggest the need for culturally tailored interventions, including community-based mental health programs, language-accessible services, social integration initiatives, and interventions addressing perceived discrimination. In conclusion, while the findings provide important insights into predictors of psychological distress among Afghan and Syrian refugee women, cross-group comparisons involving ASC symptom domains should be interpreted with caution until further cross-cultural validation studies are conducted.

## Data Availability

The raw data supporting the conclusions of this article will be made available by the authors, without undue reservation.
