# Supplementary material for: Predictors of psychological distress among Afghan and Syrian refugee women in Houston, Texas
Source: Front Glob Womens Health. 2026 Jul 9;7:1830947. doi: 10.3389/fgwh.2026.1830947 (PMC13391813; doi:10.3389/fgwh.2026.1830947)
Supplement: Supplementary file 1 [file Datasheet1.docx]

**Supplementary File**

**Supplementary Table 1: Additional Characteristics and Psychosocial Measures by Refugee Group**

| Characteristic | Total (N=131) | Syrian (n=64) | Afghan (n=67) | p-value |
| --- | --- | --- | --- | --- |
| Socio-demographics |  |  |  |  |
| Education, n (%) |  |  |  | 0.804 |
| High school | 30 (23.3%) | 16 (25.8%) | 14 (20.9%) |  |
| < High school | 60 (46.5%) | 28 (45.2%) | 32 (47.8%) |  |
| > High school | 39 (30.2%) | 18 (29.0%) | 21 (31.3%) |  |
| Employment (Yes), n (%) | 29 (22.5%) | 13 (20.6%) | 16 (24.2%) | 0.624 |
| Clinical Factors |  |  |  |  |
| Diabetes (DM), n (%) | 15 (11.5%) | 10 (15.6%) | 5 (7.5%) | 0.143 |
| Hypothyroidism, n (%) | 12 (9.2%) | 9 (14.3%) | 3 (4.5%) | 0.054 |
| Psychosocial Measures |  |  |  |  |
| Aggression: mean (SD) | 5.32 (1.80) | 5.06 (1.94) | 5.56 (1.62) | 0.115 |
| Lack of Host Country Specific Competencies: mean (SD) | 10.06 (4.29) | 10.58 (4.15) | 9.73 (4.39) | 0.374 |
| Economic Strain: mean (SD) | 8.87 (4.18) | 8.36 (3.93) | 9.19 (4.34) | 0.366 |
| Social Strain: mean (SD) | 7.72 (3.64) | 7.21 (3.03) | 8.04 (3.97) | 0.282 |
| Fishar: mean (SD) | 3.47 (1.80) | 3.50 (1.79) | 3.47 (1.82) | 0.914 |

**Supplementary Table 2. Model Fit Statistics for Multivariable Linear Regression Models**

| Outcome | Number of observations used | R² | F-statistic | p-value | Root MSE |
| --- | --- | --- | --- | --- | --- |
| Aggression | 71 | 0.319 | 2.27 | 0.0195 | 1.44 |
| Jigar Khun | 69 | 0.4879 | 4.03 | 0.0001 | 9.2818 |
| Fishar | 116 | 0.23 | 2.18 | 0.0219 | 1.79 |

**Note:** R² represents the proportion of variance explained by each model. F-statistics and corresponding p-values reflect overall model significance. Root MSE indicates model prediction error. Sample sizes vary across models due to missing data.

**Supplementary Table 3. Multicollinearity Diagnostics for the Aggression Model**

| Variable | Tolerance | VIF |
| --- | --- | --- |
| Refugee status | 0.544 | 1.84 |
| Perceived discrimination | 0.734 | 1.36 |
| Host community stress | 0.633 | 1.58 |
| Economic strain | 0.53 | 1.89 |
| Social strain | 0.51 | 1.96 |
| Age | 0.569 | 1.76 |
| BMI | 0.659 | 1.52 |
| Insurance | 0.592 | 1.69 |
| Hypertension | 0.693 | 1.44 |
| Education (High school) | 0.785 | 1.27 |
| Education (> High school) | 0.681 | 1.47 |
| Income (> $10,000) | 0.779 | 1.28 |

**Supplementary Table 4. Multicollinearity Diagnostics for the Sadness and Social Withdrawal Model (Jigar Khun)**

| Variables | Tolerance | VIF |
| --- | --- | --- |
| Refugee status  (Afghan vs Syrian) | 0.50 | 2.02 |
| Living with partner  (No vs Yes) | 0.62 | 1.62 |
| Education  High school vs> High school  <High school vs >High school | 0.59  0.47 | 1.69  2.12 |
| Family Income  <$10,000 vs >$10,000 | 0.69 | 1.44 |
| Insurance  No vs Yes | 0.50 | 2.00 |
| Household Number | 0.65 | 1.53 |
| BMI | 0.65 | 1.54 |
| Age | 0.67 | 1.50 |
| Perceived discrimination | 0.75 | 1.34 |
| Host competence | 0.57 | 1.74 |
| Economic strain | 0.52 | 1.91 |
| Social strain | 0.50 | 2.00 |

**Supplementary Table 5. Multicollinearity Diagnostics for the Ruminative Sadness Without Social Isolation Model (Fishar)**

| Predictor | Tolerance | VIF |
| --- | --- | --- |
| Refugee status (Syrian vs Afghan) | 0.69 | 1.46 |
| Perceived discrimination | 0.85 | 1.17 |
| Host country–specific competence | 0.69 | 1.44 |
| Economic strain | 0.57 | 1.75 |
| Social strain | 0.57 | 1.76 |
| Age | 0.76 | 1.31 |
| Body mass index (BMI) | 0.74 | 1.35 |
| Insurance (No vs Yes) | 0.72 | 1.39 |
| Education (> High school vs < High school) | 0.86 | 1.16 |
| Family income (>$10,000 vs <$10,000) | 0.84 | 1.19 |

**Supplementary Table 6. Internal consistency reliability of ASC and RPMS scales in the study sample (N = 131)**

| Scale / Domain | Number of Items | Cronbach’s α |
| --- | --- | --- |
| Afghan Symptoms Checklist (ASC) |  |  |
| Jigar Khun subscale | 14 | 0.905 |
| Fishar subscale | 2 | 0.590* |
| Aggression subscale | 4 | 0.409 |
| Refugee Post-Migration Stress Scale (RPMS) |  |  |
| Perceived discrimination | 4 | 0.67 |
| Lack of host country-specific competencies | 3 | 0.859 |
| Material and economic strain | 3 | 0.868 |
| Social strain | 3 | 0.705 |

* For the 2-item Fishar subscale, Spearman-Brown coefficient was additionally examined (Spearman-Brown coefficient = 0.59, p < 0.0001).

**Supplementary Table 7. Comparison of Participants Included vs Excluded from Regression and Jigar Khun Models**

| Variable | Aggression Model (p) | Jigar Khun Model (p) |
| --- | --- | --- |
| Age | 0.556 | 0.853 |
| BMI | 0.052 | 0.047 |
| Refugee Group | 0.002 | 0.896 |
| Education | 0.818 | 0.402 |
| Family Income | 0.134 | 0.693 |
| Insurance | 0.527 | 0.527 |
| Living with Partner | 0.714 | 0.714 |
